# The association between dietary protein intake and esophageal cancer risk: a meta-analysis

**DOI:** 10.1042/BSR20193692

**Published:** 2020-01-17

**Authors:** Fanjuan Kong, Erdong Geng, Juan Ning, Zhiyu Liu, Aihua Wang, Siyu Zhang, Hua Wang

**Affiliations:** 1 Hunan Provincial Maternal and Child Health Care Hospital, Changsha 410008, Hunan Province, China; 2 Department of Gastroenterology, The Cardiovascular Department of Beijing Royal Integrative Medicine Hospital, Beijing, China; 3 Department of Oncology, Huairou Hospital, Beijing Chaoyang Hospital, Capital Medical University, Beijing 101400, China; 4 Hunan Provincial Center for Disease Control and Prevention, Changsha 410008, Hunan Province, China

**Keywords:** Dietary, Esophageal cancer, Meta-analysis, Protein

## Abstract

Several papers studied dietary protein intake as a potential influence factor for esophageal cancer, but their findings were inconsistent. Thus, this meta-analysis was performed to identify the effect of protein intake on esophageal cancer risk. Potential case–control studies or cohort studies from the databases of Embase, Web of Science and PubMed were searched. The strength of association was quantified by pooling odds ratio (OR) and 95% confidence interval (CI). In total, 11 articles involving 2537 cases and 11432 participants were included in this meta-analysis. As a result, dietary protein intake had non-significant association on esophageal cancer risk overall (pooled OR = 1.11, 95% CI = 0.88–1.40). Meanwhile, we obtained consistent results in the subgroups analyses by study design, protein type, geographic locations and number of cases. Interestingly, dietary protein intake could significantly increase the risk of esophageal squamous cell carcinoma (pooled OR = 1.29, 95% CI = 1.02–1.62), instead of other disease type. To sum up, dietary protein intake had no significant association with esophageal cancer risk in the overall analysis; but, protein intake may be associated with the risk of esophageal squamous cell carcinoma. While some limitations existed in the present paper, more studies with large sample size are warranted to further confirm this result.

## Introduction

Esophageal cancer is considered as the eighth most common cancer worldwide [1]. It was estimated that there were 572034 new esophageal cancer cases in 2018 [2]. Meanwhile, it is a multifactorial disease, which may be affected by numerous genetic factors [3,4] or some environmental factors [5,6]. Furthermore, dietary factors [7] may affect the risk of esophageal cancer. Previous studies suggested that vitamins intake [8,9], fiber intake [10], folate intake [11,12], could decrease the development of esophageal cancer. Intakes of bioactive compounds from various plant sources also reduced the risk of cancer [13–16]. Previous meta-analyses had been published to assess the association between dietary protein intake and many cancers, such as prostate cancer [17], colorectal cancer [18], ovarian cancer [19] etc. However, no meta-analysis was conducted about protein intake and esophageal cancer risk. Up to now, many original articles were published regarding protein intake on esophageal cancer risk. The findings of these studies were inconclusive and inconsistent through review of the original articles. This may be attributed to the small sample sizes, heterogeneity or ethnic differences. To solve the inconsistency among these studies, we designed this meta-analysis to clarify the potential relationship about protein intake and esophageal cancer risk.

## Methods

### Literature search and inclusion criteria

Two reviewers systematically and independently searched Embase, Web of Science and PubMed to find potential studies without any restriction. The search time was from beginning to 31 July 2019. The keywords included ‘dietary’ AND ‘protein’ AND (‘esophageal cancer’ OR ‘esophageal adenocarcinoma’ OR ‘esophageal squamous cell carcinoma’). The search strategy for computerized literature search conducted in PubMed is listed in Supplementary Table S1. References of identified studies were manually screened to search any omitted articles. The inclusion criteria were (1) case–control studies or cohort studies; (2) having available odd ratios (ORs) and its 95% confidence intervals (CIs) or enough data for calculating them; (3) evaluation of the relationship about protein intake and esophageal cancer risk; (4) human studies. All papers were searched if they met our inclusion criteria no matter full-text available or not.

### Quality assessment and data isolation

The quality assessment was using the Newcastle–Ottawa Scale (NOS) [20]. Based on the inclusion criteria, two reviewers independently extracted the data of interest, including first author, year, study type, sample sizes (cases and participants), cancer type, age, protein type, assessment of dietary protein, country of origin and ORs with their 95% CIs. If data were unavailable in an article, we contacted the authors for relevant data.

### Statistical analysis

Statistical analysis was conducted by Stata 12.0 (StataCorp, College Station, U.S.A.). Pooled ORs with their 95% CIs was calculated using the independent OR and its 95% CI in each individual study [21]. Stratified analyses were also conducted. Regarding potential heterogeneity among studies, we defined significant heterogeneity at the levels *P*<0.10 or *I^2^* > 50% [22]. A random-effect model was used in the pooled analysis. The effect on heterogeneity test and the stability of results were evaluated via meta-regression [23] and sensitivity analysis by eliminating one study each time. Publication bias was tested by visually inspecting the symmetry of Begg’s funnel plot [24] and assessing Egger’s test [25]. Statistical significance was set at *P*<0.05.

## Results

### Characteristics of included articles

**Figure 1:**
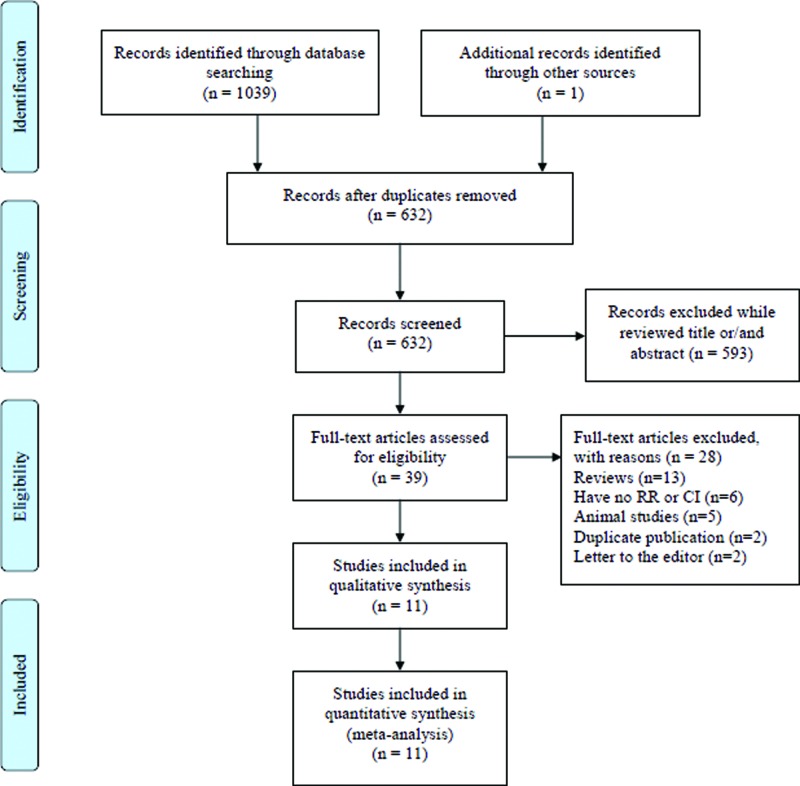
Flow chart of meta-analysis for exclusion/inclusion of studies

The initial search returned 1039 articles from the above-mentioned databases. One additional record was identified from the reference of a review. Then 408 duplicated articles from different databases were excluded, and 593 articles were omitted after title and abstract examination. Of the remaining 39 articles, full texts were reviewed. Twenty-eight articles were further excluded due to the reason present in Figure 1. Finally, 11 articles [26–36] with 2537 cases and 11432 participants were included. The characteristics of each study are listed in Table 1. The detailed quality assessment of each included study was present in Supplementary Table S2.

**Table 1:** Characteristics of the studies between dietary protein intake and the risk of esophageal cancer

Study, year	Design	Age	Participants, cases	Country	Disease type	Assessment of intake	Quality score	Category	OR (95% CI)	Adjusted for or matched for
Chen et al., 2002	PBCC	62.3 ± 12.4	573, 124	United States	Esophageal adenocarcinoma	HHHQ	7	Q4 vs. Q1	0.5 (0.3–1.0)	Age, age squared, sex, respondent type, BMI, alcohol use, tobacco use, education, family history of cancers, and vitamin supplement use
De Stefani et al., 1999	HBCC	NA	459, 66	Uruguay	Esophageal cancer	FFQ	7	Highest vs. Lowest	1.5 (1.1–2.2)	Age, sex, residence, urban/rural status, education, BMI, tobacco smoking, total alcohol intake and total energy intake
De Stefani et al., 2006	HBCC	40-89	1170, 234	Uruguay	Esophageal squamous cell carcinoma	FFQ	7	Q4 vs. Q1	1.01 (0.61–1.67)	Age, sex, residence, urban/rural status, birthplace, education, body mass index, smoking status, years since quit smoking, number of cigarettes smoked per day, alcohol drinking, mate consumption, and total energy intake
Jessri et al., 2011	HBCC	40-75	143, 47	Iran	Esophageal squamous cell carcinoma	FFQ	8	T3 vs. T1	1.93 (0.6–3.18)	Age, sex, reflux, BMI, smoking, physical activity, and education
Lagergren et al., 2013	PBCC	<80	1008, 188	Sweden	Esophageal adenocarcinoma	FFQ	8	Q4 vs. Q1	0.86 (0.51–1.45)	Age, sex, reflux, BMI, smoking, alcohol consumption, education grade, and total energy intake
Lagergren et al., 2013	PBCC	<80	987, 167	Sweden	Esophageal squamous cell carcinoma	FFQ	8	Q4 vs. Q1	1.15 (0.68–1.94)	Age, sex, reflux, BMI, smoking, alcohol consumption, education grade, and total energy intake
Mayne et al., 2001	PBCC	30-80	969, 282	United States	Esophageal adenocarcinoma	FFQ	7	T3 vs. T1	1.49 (1.02–2.18)	Age, site, sex, race, proxy status, BMI, income, education, smoking, and alcohol consumption
Mayne et al., 2001	PBCC	30–80	893, 206	United States	Esophageal squamous cell carcinoma	FFQ	7	T3 vs. T1	1.75 (1.07–2.88)	Age, site, sex, race, proxy status, BMI, income, education, smoking, and alcohol consumption
Tuyns et al., 1987	PBCC	NA	2718, 743	France	Esophageal cancer	FFQ	6	Heavy vs. Low consumers	0.51 (0.33–0.79)	Age, alcohol consumption, and tobacco smoking
Tzonou et al., 1996	HBCC	NA	256, 56	Greece	Esophageal adenocarcinoma	FFQ	7	Highest vs. Lowest	0.84 (0.56–1.27)	Age, sex, birth place, schooling, height, analgesics, coffee drinking, alcohol intake, tobacco smoking, and energy intake
Tzonou et al., 1996	HBCC	NA	243, 43	Greece	Esophageal squamous cell carcinoma	FFQ	7	Highest vs. Lowest	1.13 (0.72–1.76)	Age, sex, birth place, schooling, height, analgesics, coffee drinking, alcohol intake, tobacco smoking, and energy intake
Wolfgarten et al., 2001	PBCC	62.2 ± 1.9	140, 40	Germany	Esophageal adenocarcinoma	FFQ	8	>75 vs. <50 g/day	2.3 (0.7–6.8)	Age, gender, height, weight, BMI and socioeconomic data such as marital status and earning capacity
Wolfgarten et al., 2001	PBCC	58.1 ± 1.2	145, 45	Germany	Esophageal squamous cell carcinoma	FFQ	8	>75 vs. <50 g/day	1.7 (0.4–6.2)	Age, gender, height, weight, BMI and socioeconomic data such as marital status and earning capacity
Wu et al., 2007	PBCC	30–74	1514, 206	United States	Esophageal adenocarcinoma	FFQ	8	Q4 vs. Q1	2.22 (1.20–3.90)	Age, sex, race, birth place, education, smoking, BMI, reflux, use of vitamins, total calories, and fat
Zhang et al. 1997	HBCC	NA	214, 90	United States	Esophageal adenocarcinoma	HHHQ	7	Q4 vs. Q1	0.8 (0.6–1.2)	Age, sex, race, education, smoking, alcohol intake, BMI, and total dietary intake in calories

Abbreviations: BMI, body mass index; FFQ, food frequency questionnaire; HBCC, hospital-based case–control study; HHHQ, health habits and history questionnaire; NA, not available; PBCC, population-based case–control study; Q1, Quartile 1; Q4, Quartile 4; T1, Tertile 1; T3, Tertile 3.

### Meta-analysis results

In our included articles, four texts (Lagergren et al. (2013) [30], Mayne et al. (2001) [31], Tzonou et al. (1996) [33] and Wolfgarten et al. (2001) [34]) studied esophageal adenocarcinoma and esophageal squamous cell carcinoma at the same time. Therefore, 11 articles with 15 independent studies were used for the analysis.

**Figure 2:**
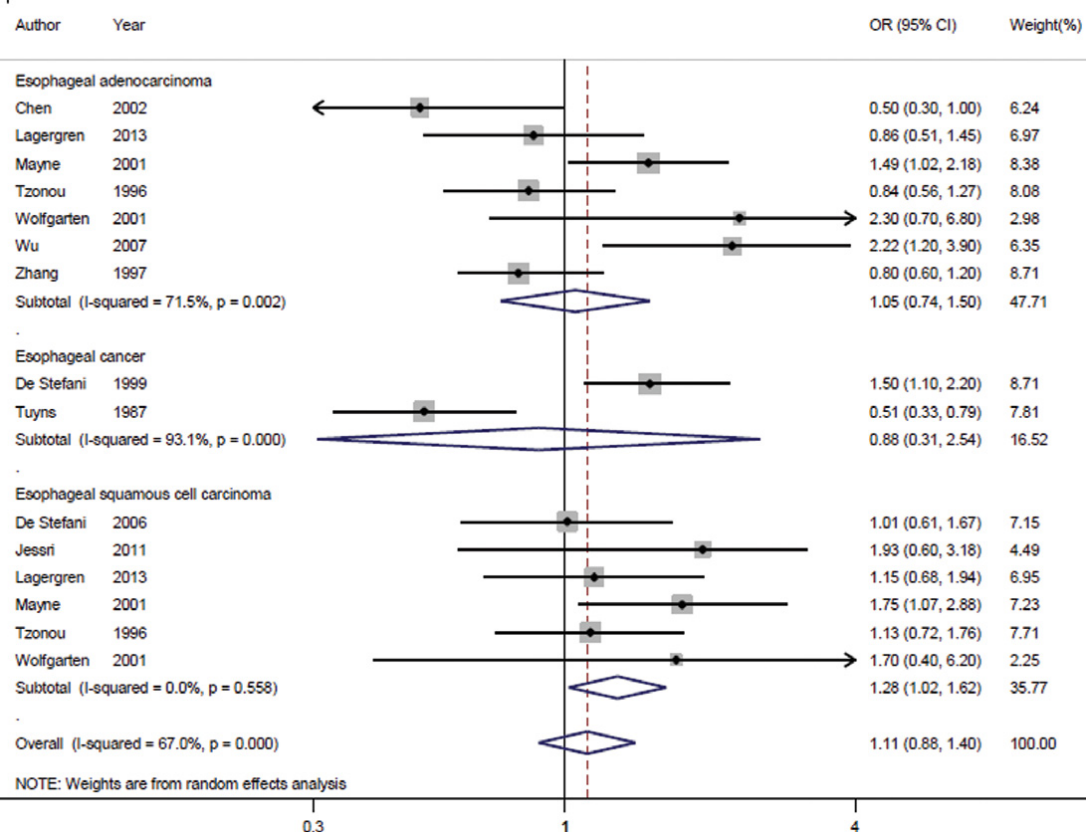
The forest plot of the association between dietary protein intake and esophageal cancer risk

Our data showed that highest category of dietary protein intake compared with lowest category had no significant association with esophageal cancer risk in the overall analysis (pooled OR = 1.11, 95% CI = 0.88–1.40, *I*^2^ = 67.0%, *P_for heterogeneity_*<0.001) (Figure 2).

Hierarchical analyses by study design, protein type (animal protein and vegetable protein), geographic locations (Europe, North America and South America) and number of cases were performed; the association was not significant in all the subgroups. Interestingly, dietary protein intake could significantly increase the risk of esophageal squamous cell carcinoma (pooled OR = 1.29, 95% CI = 1.02–1.62), instead of other disease types, when we performed the analysis between dietary protein intake and disease type (Figure 2). The detailed results are shown in Table 2.

**Table 2:** Summarized results of the protein intake and the risk of esophageal cancer

Subgroups	Number of studies	Number of cases	OR (95% CI)	*P* for trend	Heterogeneity test	
					*I^2^* (%)	*P*
Total	15	2537	1.112 (0.883–1.400)	0.367	67.0	<0.001
Disease type						
Esophageal adenocarcinoma	7	986	1.051 (0.736–1.500)	0.786	71.5	0.002
Esophageal squamous cell carcinoma	6	742	1.285 (1.019–1.620)	0.034	0.0	0.558
Study design						
PBCC	9	2001	1.142 (0.774–1.686)	0.502	75.5	<0.001
HBCC	6	536	1.080 (0.839–1.390)	0.551	48.6	0.083
Protein type						
Animal protein	5	701	1.330 (0.598–2.958)	0.484	91.7	<0.001
Vegetable protein	3	615	0.544 (0.249–1.187)	0.126	89.7	<0.001
Geographic locations						
Europe	7	1282	0.931 (0.688–1.261)	0.645	49.5	0.064
North America	5	908	1.183 (0.736–1.902)	0.486	80.9	<0.001
South America	2	300	1.286 (0.881–1.877)	0.192	37.8	0.205
Asia	1	47	-	-	-	-
Number of cases						
<200	10	866	1.041 (0.816–1.328)	0.748	51.2	0.030
≥200	5	1671	1.228 (0.741–2.036)	0.425	82.5	<0.001

Abbreviations: HBCC, hospital-based case–control study; PBCC, population-based case–control study.

### Sensitivity analyses and publication bias

**Figure 3:**
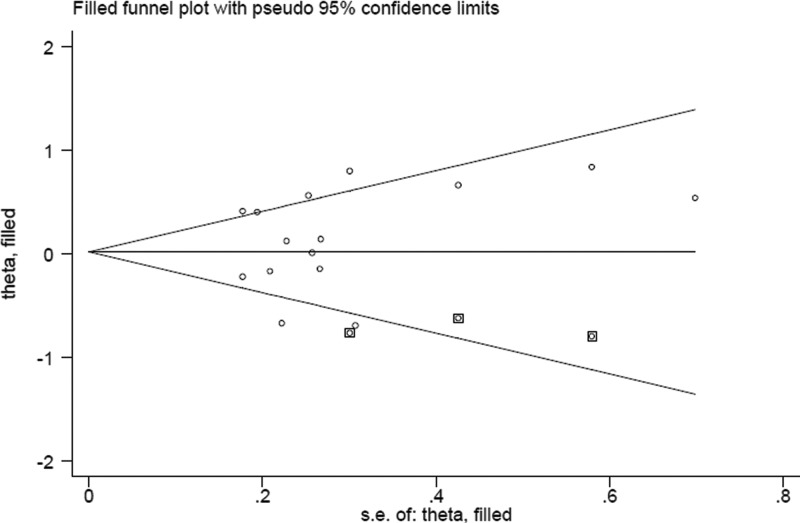
Funnel plot for the analysis of publication bias between dietary protein intake and esophageal cancer risk

Sensitivity analyses detected that no single study largely affected the overall data, indicating our results are statistically stable (data are shown in Supplementary Table S3). Neither Begg’s funnel plot (Figure 3) nor Egger’s test (*P*=0.429) found any significant publication bias.

## Discussion

Our study showed that no significant relationship was found between protein intake and esophageal cancer risk in the overall analysis, as well as in the subgroup analyses by study design, protein type, geographic locations and number of cases. However, stratified analysis of disease type showed that protein intake may be a risk factor on the development of esophageal squamous cell carcinoma, instead of esophageal adenocarcinoma.

Protein is involved in the organization of human tissues. Meanwhile, it is vital for our body’s growth and development, as well as the transport of some essential substances and the provision of bioenergy [37,38]. However, protein may affect the cancer differently with different sources [39], such as meat (red meat and processed meat), egg, soy food and milk [39]. In our study, we pooled the results for total protein intake. We only performed a subgroup analysis by animal protein and vegetable protein because no specific classification of protein intake was available in each study. Pournaghi et al. performed a study about animal protein intake with esophageal cancer risk [7]. Results from their study suggested meat intake including beef, processed meats (sausages) and chicken with skin had a positive association with esophageal cancer risk. The use of lamb meat and fish had no significant association with esophageal cancer risk [7]. Therefore, further studies with detailed sources of protein are warranted to explore some other potential results.

In a previous meta-analysis published by Mao et al. [17], dietary protein intake had no significant effects on prostate cancer risk. Pang and Wang [19] tried to assess the association about dietary protein intake and ovarian cancer risk. Similarly, they failed to find a positive result between them [19]. Results from a meta-analysis by Lai et al. [18] suggested that protein intake had no significant association with colorectal cancer risk. Our study got a consistent result with the above-mentioned meta-analyses. However, we found an increased risk on esophageal squamous cell carcinoma with high protein intake when we conducted a subgroup analysis by cancer subtypes. Different cancer pathogenesis may exist in different cancer subtypes, regarding the effect of dietary proteins [30,31].

Significant between-study heterogeneity (*I*^2^ = 67.0%, *P_for heterogeneity_*<0.001) was found in the overall results. As introduced in the ‘Methods’ section, we then used meta-regression to explore the causes of heterogeneity by the covariates of disease type, protein type, study design, geographic locations and assessment of intake. We did not find any covariates which caused this high between-study heterogeneity. Subgroup analyses were performed and the between-study heterogeneity also existed in some subgroup analyses. However, sensitivity analysis showed that no single study largely affected the overall data. Therefore, our results, in whole or in subgroup analyses, were stable.

This meta-analysis had several limitations. First, subgroup analyses of age, sex, smoking or drinking status were not conducted due to data shortage. Although we did not perform the subgroup analyses by the factors we mentioned above, most of the included studies had adjusted for age, sex, smoking or drinking status and some other related factors. Therefore, they may not affect the overall result. Second, our meta-analysis included 11 articles, which were all case–control studies. The selection bias, recall bias and some other confounding factors cannot be excluded in the case–control studies. Therefore, some cohort studies should be conducted to further confirm this result. Third, different protein types may have different effects on esophageal cancer risk. However, most of the included articles did not report the protein type with esophageal cancer risk, respectively. Fourth, dose–response analysis was not done because of the limited data in each study. Dose–response analysis would use the detailed amount of dietary protein, detailed cases and controls in each category, however, only one study (Wolfgarten et al. (2001) [34]) met the criterion of dose–response analysis. Therefore, further studies with detailed amounts of protein intake, detailed cases and controls in each category are warranted to get a dose–response result. Fifth, patients with esophageal cancer may not follow a ‘healthy diet’, such as high vegetable, fruit etc. This may increase some between-study heterogeneity and publication bias. At last, all the included articles were with English language. This may omit some articles which were with other languages. However, no publication bias was detected in our study.

## Conclusion

In conclusion, dietary protein intake had no significant association on esophageal cancer risk in the overall analysis; but, protein intake may be associated with the risk of esophageal squamous cell carcinoma. While some limitations exited in the present paper, more studies with large sample size are warranted to further confirm this result.

## Supplementary Material

Supplementary Tables S1-S3

## Abbreviations

CI: confidence interval
OR: odds ratio
