# Supplementary material for: The association between dietary protein intake and esophageal cancer risk: a meta-analysis
Source: Biosci Rep. 2020 Jan 17;40(1):BSR20193692. doi: 10.1042/BSR20193692 (PMC6970082; doi:10.1042/BSR20193692)
Supplement: Supplementary Tables S1-S3 [file BSR-2019-3692_supp.pdf]

Supplementary table 1 Search criteria for computerized literature search conducted in PubMed.

Search	Search terms and combinations
1	(“diet” [Text Word] OR “dietary” [Text Word])
2	“protein” [Text Word]
3	("esophageal cancer" [Text Word] OR "esophageal adenocarcinoma" [Text Word] OR "esophageal squamous cell carcinoma" [Text Word])
4	1 AND 2 AND 3

Supplementary table 2 Detailed information about quality assessment in each included study.

Study	Selection(****)	Comparability(**)	Exposure(***)	Total
Chen et al. 2002	****	*	**	7
De Stefani et al. 1999	***	**	**	7
De Stefani et al. 2006	***	*	***	7
Jessri et al. 2011	****	*	***	8
Lagergren et al. 2013	***	**	***	8
Mayne et al. 2001	***	*	***	7
Tuyns et al. 1987	***	*	**	6
Tzonou et al. 1996	***	**	**	7
Wolfgarten et al. 2001	****	**	**	8
Wu et al. 2007	****	*	***	8
Zhang et al. 1997	***	*	***	7

Supplementary table 3 Sensitivity analyses about protein intake and risk of esophageal cancer.

Study omitted	Disease type	OR(95%CI)
Chen et al. 2002	Esophageal adenocarcinoma	1.17(0.93-1.46)
De Stefani et al. 1999	Esophageal cancer	1.08(0.85-1.38)
De Stefani et al. 2006	Esophageal squamous cell carcinoma	1.12(0.88-1.44)
Jessri et al. 2011	Esophageal squamous cell carcinoma	1.08(0.86-1.37)
Lagergren et al. 2013	Esophageal adenocarcinoma	1.14(0.89-1.45)
Lagergren et al. 2013	Esophageal squamous cell carcinoma	1.11(0.87-1.42)
Mayne et al. 2001	Esophageal adenocarcinoma	1.08(0.85-1.38)
Mayne et al. 2001	Esophageal squamous cell carcinoma	1.07(0.85-1.36)
Tuyns et al. 1987	Esophageal cancer	1.18(0.95-1.46)
Tzonou et al. 1996	Esophageal adenocarcinoma	1.14(0.89-1.46)
Tzonou et al. 1996	Esophageal squamous cell carcinoma	1.11(0.87-1.43)
Wolfgarten et al. 2001	Esophageal adenocarcinoma	1.09(0.86-1.37)
Wolfgarten et al. 2001	Esophageal squamous cell carcinoma	1.10(0.87-1.39)
Wu et al. 2007	Esophageal adenocarcinoma	1.06(0.84-1.33)
Zhang et al. 1997	Esophageal adenocarcinoma	1.15(0.90-1.47)
Combined		1.11(0.88-1.40)
